# Forced expiratory volume in one second: A novel predictor of work disability in subjects with suspected obstructive sleep apnea

**DOI:** 10.1371/journal.pone.0201045

**Published:** 2018-07-19

**Authors:** Mariarita Stendardo, Valeria Casillo, Michela Schito, Licia Ballerin, Francesco Stomeo, Emanuela Vitali, Marco Nardini, Elisa Maietti, Piera Boschetto

**Affiliations:** 1 Department of Medical Sciences, University of Ferrara, Ferrara, Italy; 2 Respiratory Unit, University-Hospital of Ferrara, Ferrara, Italy; 3 Ear, Nose and Throat & Audiology Department, University-Hospital of Ferrara, Ferrara, Italy; 4 Department of Prevention and Protection, University-Hospital and Public Health Service of Ferrara, Ferrara, Italy; 5 Center for Clinical and Epidemiological Research, University-Hospital of Ferrara, Ferrara, Italy; Charite Medical University Berlin, GERMANY

## Abstract

Whether the association of work disability with obstructive sleep apnea (OSA) is mainly due to the disease, i.e. the number and frequency of apneas-hypoapneas, or to coexisting factors independent from the disease, is not well-established. In this study, we aim to evaluate work ability in a group of subjects undergoing OSA workup and to identify the major contributors of impaired work ability. In a cross-sectional study, we enrolled 146 consecutive subjects who have been working for the last five years and referred to the sleep disorders outpatients’ clinic of the University-Hospital of Ferrara, Italy, with suspected OSA. After completing an interview in which the Work Ability Index (WAI) and the Epworth Sleepiness Scale (ESS) questionnaires were administered to assess work ability and excessive daytime sleepiness, respectively, subjects underwent overnight polysomnography for OSA diagnosing and spirometry. Of the 146 subjects, 140 (96%) completed the tests and questionnaires and, of these, 66 exhibited work disability (WAI < 37). OSA was diagnosed (apnea-hypopnea index ≥ 5) in 45 (68%) of the 66 subjects. After controlling for confounders, a lower level of forced expiratory volume at 1 second (FEV_1_), [odds ratio 0.97 (95% CI 0.95–1.00)], older age [1.09 (95% CI 1.03–1.15)], excessive daytime sleepiness [3.16 (95% CI 1.20–8.34)] and a worse quality of life [0.96 (95% CI 0.94–1.00)], but not OSA [1.04 (95% CI 0.41–2.62)], were associated with work disability. Patients with a higher number of diseases, in which OSA was not included, and a lower quality of life had an increased probability of absenteeism in the previous 12 months. In subjects with suspected OSA, FEV_1_ can be an important predictor of work disability.

## Introduction

Obstructive sleep apnea (OSA), a respiratory disorder characterized by recurrent upper airway obstruction during sleep, is common among middle-aged employees [1], and its impact on public health has long been reported [2, 3]. Not only do patients with OSA deplete more health-care funds, but they also have high non-health-care-related costs principally due to work disability [4]. As previously reported by Allen AJMH et al. [5], “work disability is commonly defined by two components: work impairment, i.e. decreased performance while at work, and absenteeism which usually refers to the number of missed days or hours of work for employed people”.

Although an association between OSA and both work impairment and absenteeism have often been described [5], there is emerging evidence that factors other than the disease, and not the disease itself defined as a number of apnea-hypoapnea episodes ≥ 5 in an overnight polysomnography [6], are implicated in work disability.

Excessive daytime sleepiness (EDS), albeit considered a consequence of OSA, is present in a maximum of 50% of patients with OSA and is a strong risk factor of work-impairment, regardless of OSA [7].

OSA is usually accompanied by other diseases of which the most frequent are obesity, cardiovascular illnesses, particularly systemic arterial hypertension, and depression [5]. Obesity and OSA are exceptionally common health problems that frequently coexist, and obesity is *per se* a risk factor for exit from paid employment through disability pension [8], and strongly associated with absenteeism and overall work impairment [9]. Likewise, hypertension, that co-occurs often with OSA, is strictly related to work absences [10]. Depression is a major cause of work disability [11, 12], and it had been observed as so closely associated with OSA [13] that depressive symptoms have been considered OSA clinical features [14]. However, recently, the causal relationship between the two entities has been challenged as OSA is not a risk factor for incident hospitalized depression [15]. Although this finding has been shown to be proved for severe depression requiring hospitalization and cannot be extended to more common milder forms relating to non-restorative sleep or residual sleepiness, work disability attributed to OSA may be due to depression, at least when the latter is connected to hospital admissions. Having said that, illnesses and co-morbidities, such as obesity, hypertension and psychiatric disorders, associated frequently with OSA may be responsible, at least in part, of work impairment and absenteeism ascribed to OSA. Adequate controls of these confounders are warrant in studies aimed to evaluate the OSA contribution to work disability.

Lung function assessment should be recommended in subjects with suspected OSA to highlight obstructive lung diseases [16], overlooked respiratory conditions associated with obesity [17], and to better characterize OSA when established [18]. Furthermore, forced expiratory volume in one second (FEV1), an index utilized for assessing both obstructive and restrictive lung disorders, has recently been revealed a promising tool in general health assessment [19] and helpful in occupational settings [20]. However, spirometry has not been performed in the majority of studies on OSA conducted so far.

We, therefore, investigated the possible association between OSA and work disability in subjects in employment by using a work ability questionnaire which encompasses the registration of the impact of illnesses other than OSA on work outcomes, and by including lung function measurements.

## Methods

### Study subjects

In this cross-sectional study we enrolled patients with respiratory sleep complaints referred to the Center for Sleep Disorders and ENT (ear, nose and throat), otolaryngology and audiology services, University-Hospital of Cona (FE), Italy, for clinical suspicion of OSA by a general practitioner or a medicine specialist [21]. Patients with non-respiratory sleep complaints (e.g. narcolepsy, periodic limb movement disorder) were evaluated by another department and, therefore, not represented in our patient population.

**Fig 1:**
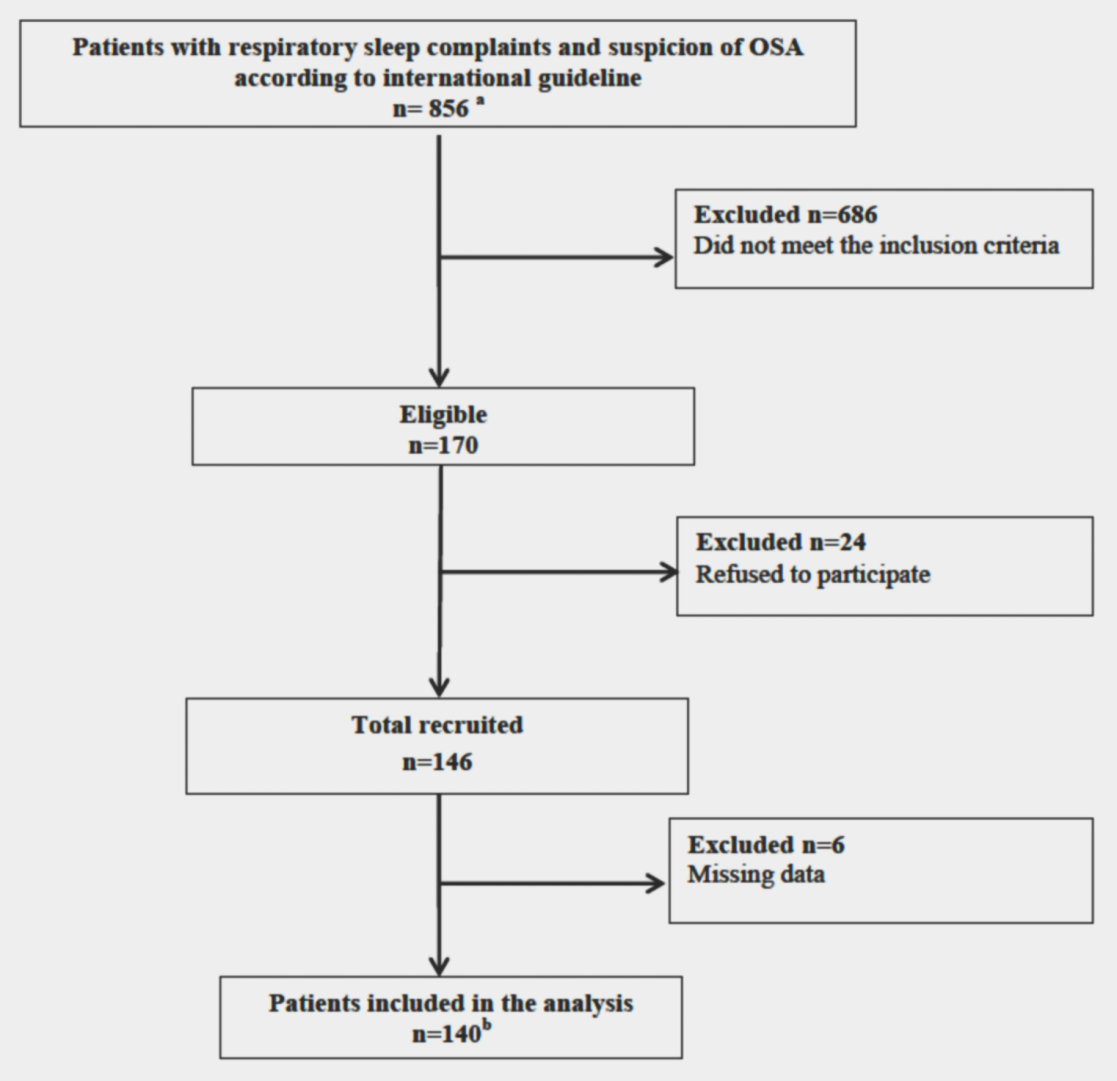
Flow chart of participants included for analysis. ^a^ Patients sent to our Sleep Disorders Clinics by general practitioner and medicine specialists for suspected OSA according to international guideline (*J Clin Sleep Med* 2009; 15: 263–76). ^b^ All the 140 patients complained symptoms: excessive daytime sleepiness (n = 45, 32%) and/or usual snoring (n = 112, 80%) and/or witnessed or perceived apnea (n = 76, 54%).

Amongst the new patients referred to our Centers from February 2015 to December 2016 (n = 856), consecutive patients between 18 and 65 years old in employment for the previous 5 years were invited to participate in the study (n = 170). Of the initial 170 eligible patients, 24 refused and 146 were recruited, but 6 were excluded from the analysis because of lack of data (Fig 1).

The study conformed to the Declaration of Helsinki and was approved by the institutional ethics committee of University-Hospital of Ferrara. All participants provided signed informed consent before recruitment (ClinicalTrials.gov number, N. 150498).

### Study design

All participants underwent clinical evaluation with data collection on demographic/clinical parameters [age, body mass index (BMI; Kg/m2), heart rate, blood pressure], work history (job type, sickness absence days, occupational injuries), smoking status (current smoker, ex-smoker, never smoker, pack-years) and presence of symptoms (common snoring, daytime sleepiness, nighttime shortness of breath perceived). Daytime sleepiness was evaluated by the Epworth Sleepiness Scale and an overnight polysomnography was performed [22].

Standardized surveys were used to measure work capacity [Work Ability Index (WAI)], quality of life [visual analogic well-being scale (VAWS)], and comorbidities [Charlson-comorbidity Index (CI)]. Finally, subjects underwent spirometry for lung function assessment.

### Epworth Sleepiness Scale

For each item in a simple self-administered eight-item questionnaire, subjects expressed a range of numeric values, from 0 to 3, thus obtaining a total score ranging from 0 (no daytime sleepiness) to 24 (the highest level of daytime sleepiness). Epworth Sleepiness Scale **(**ESS) scores of over 10 were considered to indicate excessive daytime sleepiness [22].

### Work Ability Index

Work ability was measured using the WAI, which has been shown to be a valid, reliable, and cross-national instrument for use in occupational health [23]. The WAI consists of 7 dimensions. Dimension 1 asks to indicate on a scale from 0 (not able to work) to 10 (lifetime best) to estimate the current work ability compared with the lifetime best. Dimension 2 contains two questions and assesses on a 5-point scale ranging from 1 (very poor) to 5 (very good) the subjective current work ability in relation to the physical and mental demands of work (sum score of dimension 2 ranges from 2–10). Dimension 3 registers the number of diseases diagnosed by a physician. Dimension 4 assesses on a six point scale ranging from 1 (fully impaired) to 6 (no impairments) the subjective estimation of work impairments due to disease by asking whether the illness is a hindrance in their job. Dimension 5 concerns the number of days off work due to sick leave in the previous 12 months, with answering categories ranging from 0 days (5 points) to 100 days or more (1 point). We categorized the total number of sick days during the preceding year into 3 classes: 0 days, 0<days≤9 and > 9 days. Dimension 6 asks ‘Do you believe, according to your present state of health, that you will be able to do your current job two years from now?’: a score of 1 (hardly able to work), 4 (not sure), or 7 (fairly sure) could be obtained. Dimension 7 assesses the mental resources in the past few months using three questions concerning enjoying regular daily activities, being active and alert, and feeling to be full of hope about the future, with answering categories ranging from ‘never’ (0 points) to ‘always’ (4 points). For dimension 7 a sum score was calculated, leading to a score of 1 (if the sum score ranged between 0–3 points) to 4 (if the sum score ranged between 10–12 points). The total WAI score was calculated as the sum score of the 7 dimensions and ranges from 7–49. The WAI was categorized into “poor” (score 7–27), “moderate” (score 28–36), “good” (score 37–43), and “excellent” (score 44–49) work ability. For our analysis, we aggregated poor and moderate work ability into one category (WAI < 37) and good and excellent work ability (WAI ≥ 37) into another [24].

### Visual analogic well-being scale

The visual analogic well-being scale is a simple and rapid test to evaluate the health-related quality-of-life (HRQL). It consists of a 120-mm straight line on which the patient indicates his or her health status with respect to the symptoms which were the motive for the consultation. The extremes of the line indicate the least and the most favorable well-being status. The distance (in mm) between the point marked by the patient and the least favorable position is measured and transformed into a percentage [25].

### Charlson index

The Charlson is probably the most widely used comorbidity index to date. CI is a valid test for predicting the outcome and risk of death from many comorbid diseases. It is calculated according to the scoring system established by Charlson et al. and is considered the gold standard for the assessment of comorbidity risk in clinical research [26].

### Polysomnography

The diagnosis of OSA was established by overnight polysomnography. Measured variables included oronasal flow by nasal cannula, thoracoabdominal movements and pulse oximetry. Apnea was defined by the absence of airflow for at least 10s. Hypopnoeas were characterized as any airflow reduction of ≥30% that lasted for ≥10s and resulted in either arousal or oxyhemoglobin desaturation. An oxyhemoglobin desaturation event was a decrease in arterial oxygen saturation of ≥4%. The apnea-hypopnea index (AHI) was obtained by the sum of the numbers of apneas and hypopneas per hour of sleep.

According to established guidelines, patients were classified as having OSA if the AHI was ≥ 5 [6]. We analyzed the association of OSA with work disability by dichotomized subjects as either meeting or failing to meet criteria for OSA (AHI ≥ 5 or AHI < 5).

### Spirometry

Forced expiratory volume (FEV_1_), forced vital capacity (FVC) and the FEV_1_/FVC ratio were measured using a spirometer (Biomedin, Padova, Italy), and the best of three values was expressed as a percentage of the predicted normal value. All measurements were obtained and interpreted in accordance with the recommendations of the American Thoracic Society/European Respiratory Society (ATS/ERS, 2005) [27].

### Statistical analysis

Continuous data were summarized using mean and standard deviation or with median and inter-quartile range as appropriate; comparisons between groups were done using t-test on means or with the non parametric Wilcoxon Mann Whitney test, according to variables distribution. Categorical variables where instead presented as frequencies and percentages and comparisons were performed using the Pearson’s chi squared test or Fisher’s exact test.

Simple and multiple logistic regression models were then estimate in order to detect the independent significant risk factors for the outcome poor-moderate working ability and work absences > 9 days. Odds Ratios (OR) and their 95%CI were reported. Candidate variables to be included in the logistic model were selected on the basis of previous comparison analysis and of clinical plausibility as risk factors, excluding redundant variables.

All analyses were done using STATA 13.0 for Windows (Stata Corporation, College Station, TX) and a p-value lower than 0.05 was considered statistically significant.

## Results

### Baseline characteristics

The baseline characteristics of the 140 study participants are shown in Table 1. Of these, 104/140 (75%) were male and the mean age (± SD) was 51 years old (±9). With regards to the symptom distribution (common snoring, daytime sleepiness, nighttime shortness of breath perceived), 80% of patients had regular snoring (> 2 times a week), and more than 50% had at least one episode of regular nighttime shortness of breath perceived. 32% of subjects suffered from daytime sleepiness (ESS>10), the symptom occurring in about 1 patient out of 3.

**Table 1:** Baseline characteristics of subjects with suspected obstructive sleep apnea (OSA).

	Complete sample
	(n = 140)
**Age (years)**	51 ± 9
**Males (n (%))**	104 (74)
**Smoking history (n (%))**	
• Never	57 (41)
• Ex/Current	83 (59)
**Pack years (median (IQ range))**	5 (0–16)
**BMI (Kg/m**^**2**^**)**	30.6 ± 7.4
**Symptom (n (%))**	
• Usual snoring (>2/week)	112 (80)
• Nighttime shortness of breath perceived	76 (54)
• Excessive Daytime Sleepiness (ESS>10)	45 (32)
**OSA (n (%))**	83 (59)
**AHI**	20 ± 25
**Hypertension (n (%))**	60 (43)
**Depression (n (%))**	3 (2)
**Charlson index (median (IQ range))**	1 (0–2)
**Spirometry**	
• FEV_1_% Predicted	100 ± 18
• FEV_1_/FVC	79.2 ± 8.5
**VAWS**	58 ± 23
**Work related injuries (n (%))**	36 (26)
**Manual work (n (%))**	87 (62)
**WAI**	35 ± 7
**Work absences (>9 days) (n (%))**	40 (29)

Data are expressed as number of subjects (%) or mean ± SD or median (IQ range).

Abbreviations: BMI, body mass index; OSA, obstructive sleep apnea; AHI, apnea-hypopnea index; FEV_1_, forced expiratory volume in 1 second; FVC, forced vital capacity; VAWS, visual analogic well-being scale; WAI, work ability index.

More than half of the patients examined (59%) had OSA, 16 (11%) suffered from chronic obstructive pulmonary disease (COPD), defined according to GOLD guidelines, and 8 (11%) had both diseases [28].

The mean WAI score was 35 and 66 subjects exhibited work disability (WAI <37). Of these, 46 (33%) were in the moderate and 20 (14%) in the poor category. The other 74 subjects with WAI≥37 were: 14 (10%) in the excellent and 60 (43%) in the good class.

Forty of the total subjects (29%) had been absent more than 9 days in the last year, 48 (34%) subjects did not have any days off and 52 (37%) had up to 9 days of absences.

In a separate analysis we evaluated the characteristics of the OSA population: mean AHI was 20 events·h−1 with 17% of patients suffering from a mild (AHI 5–15 events per hour), 18% a moderate (15–30 events per hour) and 24% a severe (more than 30 events per hour) disease. Subjects with OSA were significantly older, mostly males and had a higher BMI than those without OSA. Between the OSA and no OSA the pack years and the smoking history distribution were similar as well as the spirometry data. The OSA had a higher Charlson index (which did not include OSA) and a higher percentage of subjects suffering of hypertension. The median value of WAI was significantly lower in OSA, whilst no differences were found in day work absences (Table 2).

**Table 2:** Baseline characteristics of subjects with and without obstructive sleep apnea (OSA).

	OSA	NO OSA	P-value
	(n = 83)	(n = 57)	
**Age (years)**	53 ± 8	48 ± 9	0.001
**Males (n (%))**	67 (81)	37 (65)	0.035
**Smoking history (n (%))**			
Never	31 (37)	26 (46)	0.568
Ex/Current	52 (63)	31 (54)	
**Pack years (median (IQ range))**	6 (0–20)	3 (0–10)	0.145
**BMI (Kg/m**^**2**^**)**	32.7 ± 7.9	27.6 ± 5.3	<0.001
**Symptom (n (%))**			
• Usual snoring (>2/week)	71 (85)	41 (72)	0.048
• Nighttime shortness of breath perceived	51 (61)	25 (44)	0.040
• Excessive Daytime Sleepiness (ESS>10)	27 (32)	18 (32)	0.906
**Hypertension (n (%))**	44 (53)	16 (28)	0.003
**Depression (n (%))**	2 (3)	1(2)	0.632
**Charlson index (median (IQ range))**	1 (0–2)	0 (0–1)	<0.001
**Spirometry**			
• FEV_1_% Predicted	100 ± 20	101 ± 16	0.784
• FEV_1_/FVC	79.9 ± 8.6	78.2 ± 8.4	0.253
**VAWS**	54 ± 25	64 ± 19	0.008
**Manual work (n (%))**	57 (69)	30 (53)	0.055
**Work related injuries (n (%))**	25 (30)	11 (19)	0.150
**WAI**	34.1 ± 7.5	37.6 ± 6.2	0.004
**Work absences (>9 days) (n (%))**	24 (28.9)	16 (28.1)	0.913

Data are expressed as number of subjects (%) or mean ± SD or median (IQ range).

Abbreviations: OSA, obstructive sleep apnea; BMI, body mass index; FEV_1_, forced expiratory volume in 1 second; FVC, forced vital capacity; VAWS, visual analogic well-being scale; WAI, work ability index.

### Work disability and day work absences >9 days

Table 3 shows the baseline characteristics of the population according to the presence or absence of work disability: subjects with WAI<37 and WAI≥37. The former were older and had a greater BMI while the smoking history, pack-years and the percentage of males and females were similar in the two groups.

**Table 3:** Baseline characteristics according to subjects with poor-moderate (WAI<37) and good-excellent (WAI≥37) work ability.

	WAI<37	WAI ≥ 37	P-value
	(n = 66)	(n = 74)	
**Age (years)**	53.6 ± 8.3	48.7 ± 8.9	0.001
**Males (n (%))**	47 (71)	57 (77)	0.432
**Smoking history (n (%))**			
• Never	23 (35)	34 (46)	0.182
• Ex/Current	43 (65)	40 (54)	
**Pack years (median (IQ range))**	7.5 (0–20)	1.4 (0–13)	0.057
**BMI (Kg/m**^**2**^**)**	33.2 ± 8.5	28.3 ± 5.4	<0.001
**Symptom (n (%))**			
• Usual snoring (>2/week)	52 (79)	60 (81)	0.735
• Nighttime shortness of breath perceived	43 (65)	33 (45)	0.015
• Excessive Daytime Sleepiness (ESS>10)	30 (45)	15 (20)	0.001
**OSA (n (%))**	45 (68)	38 (51)	0.043
**Hypertension (n (%))**	36 (55)	24 (32)	0.008
**Depression (n (%))**	3 (4)	0 (0)	0.056
**Charlson index (median (IQ range))**	1 (1–2)	0 (0–1)	<0.001
**Spirometry**			
• FEV_1_% Predicted	95 ± 21	105 ± 14	0.001
• FEV_1_/FVC	78.4 ± 9.8	79.9 ± 7.2	0.314
**VAWS**	47 ± 22	68 ± 20	<0.001
**Manual work (n (%))**	49 (74)	38 (51)	0.005
**Work related injuries (n (%))**	22 (33)	14 (19)	0.051

Data are expressed as number of subjects (%) or mean ± SD or median (IQ range).

Abbreviations: WAI, work ability index; BMI, body mass index; OSA, obstructive sleep apnea; FEV1, forced expiratory volume in 1 second; FVC, forced vital capacity; VAWS, visual analogic well-being scale.

Patients with work disability suffered hypertension (55%), excessive daytime sleepiness (45%) and OSA (68%) and had a higher CI score. VAWS questionnaire score and FEV_1_% predicted values were both lower in subjects with WAI<37.

Table 4 shows the baseline characteristics of the population based on subjects with day work absences of ≤ 9 or >9 days. The group with fewer days of absence had better FEV_1_ and CI, a better VAWS score, a lower percentage of manual workers and of work related injuries.

**Table 4:** Baseline characteristics according to subjects with work absences ≤ 9 days and > 9 days.

	Work absences ≤ 9 days	Work absences > 9 days	P-value
	(n = 100)	(n = 40)	
**Age (years)**	50.8 ± 9.1	51.6 ± 8.7	0.628
**Males (n (%))**	75 (75)	29 (73)	0.760
**Smoking history (n (%))**			
• Never	43 (43)	14 (35)	0.384
• Ex/Current	57 (57)	26 (65)	
**Pack years (median (IQ range))**	4 (0–15)	8 (0–20)	0.293
**BMI (Kg/m**^**2**^**)**	30.3 ±7.3	31.4 ± 7.7	0.414
**Symptom (n (%))**			
• Usual snoring (>2/week)	82 (82)	30 (75)	0.350
• Nighttime shortness of breath perceived	52 (52)	24 (60)	0.391
• Excessive Daytime Sleepiness (ESS>10)	28 (28)	17 (43)	0.097
**OSA (n (%))**	59 (59)	24 (60)	0.913
**Hypertension (n (%))**	38 (38)	22 (55)	0.066
**Depression (n (%))**	3 (3)	0 (0)	0.259
**Charlson index (median (IQ range))**	0 (0–1)	1 (1–2)	**<0.001**
**Spirometry**			
• FEV_1_% Predicted	103 ± 18	94 ± 18	**0.018**
• FEV_1_/FVC	79.4 ± 8.9	78.7 ± 7.6	0.687
**VAWS**	63 ± 21	46 ± 23	**<0.001**
**Manual Work (n (%))**	57 (57)	30 (75)	**0.047**
**Work related injuries (n (%))**	20 (20)	16 (40)	**0.014**

Data are expressed as number of subjects (%) or mean ± SD or median (IQ range).

Abbreviations: BMI, body mass index; OSA, obstructive sleep apnea; FEV_1_, forced expiratory volume in 1 second; FVC, forced vital capacity; VAWS, visual analogic well-being scale.

### Univariate and multivariate analysis of factors associated with outcomes

Table 5 shows the association between work disability (WAI<37) and clinical and occupational features in the study population. All the diseases diagnosed by a physician, with the exception of OSA, were excluded from this analysis as they were an integral part of the WAI questionnaire (Dimension 3). In the simple linear regression model, WAI<37 was associated with age, OSA, symptoms (nighttime shortness of breath perceived and excessive daytime sleepiness), manual work, work related injuries, FEV_1_ and VAWS. The multivariate model, adjusting for the same potential confounders, confirmed the associations of age, excessive daytime sleepiness, FEV_1_ and VAWS.

**Table 5:** Logistic regression analyses of the association between poor-moderate work ability (WAI<37) and the clinical and occupational features of the study population (n = 140).

WAI<37	Univariate OR (95%CI)	p-value	Multivariate OR (95%CI)	p-value
**OSA**	2.03 (1.02–4.05)	**0.044**	1.04 (0.41–2.62)	0.931
**Age**	1.07 (1.03–1.11)	**0.002**	1.09 (1.03–1.15)	**0.006**
**Male**	0.74 (0.35–1.58)	0.433	0.49 (0.17–1.42)	0.190
**Excessive Daytime Sleepiness**	3.28 (1.56–6.91)	**0.002**	3.16 (1.20–8.34)	**0.022**
**Nighttime shortness of breath perceived**	2.32 (1.17–4.60)	**0.016**	1.95 (0.83–4.61)	0.128
**FEV**_**1**_**% Predicted**	0.97 (0.95–0.99)	**0.002**	0.97 (0.95–1.00)	**0.040**
**Manual work**	2.73 (1.33–5.59)	**0.006**	1.77 (0.70–4.48)	0.231
**Work related injuries**	2.14 (0.99–4.65)	**0.054**	2.59 (0.92–7.32)	0.073
**VAWS**	0.95 (0.94–0.97)	**<0.001**	0.96 (0.94–1.00)	**0.001**

Data are expressed as odds ratio (OR) and confidence intervals (CI).

Abbreviations: WAI, Work Ability Index; OSA, obstructive sleep apnea; FEV_1_, forced expiratory volume in 1 second; VAWS, visual analogic well-being scale.

FEV_1_ was shown to be a predictive factor of work disability both in the group of OSA [OR = 0.98 (95% CI: 0.95–1.00, p = 0.05)] and in that without OSA [OR = 0.95 (95% CI: 0.91–0.99, p = 0.02)].

In the univariate analysis for day work absences >9 days, an association with manual work, CI score, FEV_1_ and VAWS score was noted. The multivariate model confirmed only the associations with the Charlson index and the visual analogic well-being scale score.

## Discussion

In this study, it was found that excessive daytime sleepiness, age and FEV_1_ are factors affecting work disability, regardless of OSA diagnosis. Patients with a higher number of diseases (OSA not included) show an increased risk of absenteeism in the previous 12 months. Furthermore, a lower quality of life impacts on both work disability and the number of days of absence.

Our finding of a relationship between EDS and work disability, irrespective of OSA diagnosis, is in agreement with the results of previous studies. Mulgrew et al. found a strong association between subjective sleepiness (as assessed by ESS) and work limitation in patients undergoing full-night polysomnography for the investigation of sleep-disordered breathing [29]. Furthermore, in their whole population, they found no significant relationship between OSA severity and decreased work productivity. In a study on the burden of illness of EDS in a heterogeneous American population, the presence of excessive sleepiness as a symptom of OSA, depression, narcolepsy, multiple sclerosis, or shift work was found to have a negative incremental impact on work productivity greater than the impairment associated with these conditions [30].

In the present workers, age is associated with an increased risk of work disability. The relationship between older age and poor work ability, as defined by WAI, has already been extensively reported in a systematic review including studies on work ability published from 1985 to 2006 identified through a structured search in Pub Med and Web of Science [24]. More recently age, together with diagnosed diseases, were proved to be the most effective factors of WAI in a population of hospital nurses [31]. Our results confirm and extend these findings to subjects referred to clinics for sleeping problems.

While the observations that excessive sleepiness and older age influence work disability were not surprising, the importance of FEV_1_ was not equally expected. Our results suggest that loss in FEV_1_ is an important factor affecting work ability, regardless of the presence of OSA disease and other risk factors, such as tobacco smoke and body mass index. FEV_1_ is the most frequently utilized index for assessing airway obstruction but, recently, has been revealed as a promising tool in general health assessment [19]. Moreover, in the last few years, the spirometry data has proved to be useful in occupational settings, given that FEV_1_ has been identified as a predictor of overall work disability in a population with different disorders [32]. To our knowledge, our study is the first that considers the effects of FEV_1_ on the work ability index in a population of subjects referred for sleeping problems.

The majority of our patients with poor or moderate work ability suffer from OSA. Nevertheless, the disease *per se* is not an independent risk factor for work disability after major confounders control. These findings support the results of other studies that have shown a weak effect of OSA on work disability when OSA was examined without respect to excessive daytime sleepiness [28,7]. By contrast, our results may appear to conflict with those of Accattoli et al. [33], who found a lesser work productivity in workers with OSA as compared to non-apneic controls. However, they did not consider the effect of sleepiness, a cardinal symptom of OSA recognized as an important risk factor for the reduction of work ability [34]. In the population of subjects who referred to our Sleep Disorder Centers, sleepiness was quite common in the no-OSA group (32%) too, but its etiology remains unclear. One possible explanation could be that it is due to disorders, other than OSA, associated with EDS such as obesity, overweight and depression. Other sleep diseases, related to excessive daytime sleepiness, such as insomnia, narcolepsy, periodic limb movement disorders and restless legs syndrome, are less probable in our sample as patients suspected of these disorders are evaluated by other departments in our University-Hospital. In any case, our results further highlight the important impact of the symptom “daytime sleepiness” on the work performance, regardless of the underlying conditions [34].

We are not aware of any previous studies evaluating work disability through the WAI questionnaire among subjects with symptoms and/or diagnosis of OSA. Measuring work ability is a complex task. WAI is a simple, validated instrument used in occupational health care and research [35]. A strength of this questionnaire is given by the fact that its index encompasses the impact of the diseases the worker is affected by, other than OSA, and so guarantees the control of these important confounders. This is extremely useful as the link between work disability and diseases like obesity, cardiovascular illnesses, particularly systemic arterial hypertension, and depression, which are frequently associated with OSA, has been previously documented [9,10,11].

We also evaluated potential risk factors for work absences > 9 days. Our results show that patients with a higher number of diseases, OSA not included, had an increased risk of absenteeism in the previous 12 months. The data obtained support prior findings showing an association between sickness absence days and the presence of ≥2 chronic diseases in 514 Italian employees [36]. Likewise, Ubalde-Lopez found that the greater the number of health conditions, the higher the risk of sickness absence in a cross sectional study of 72.370 Spanish workers [37]. Sundstrup E et al elaborate on preceding data by showing that the number of chronic diseases is progressively associated with the risk of sickness absence days. They conclude highlighting the need for initiatives targeting rehabilitation and prevention of multimorbidity among the general working population [38].

Finally, what impacts both on work disability and days of absence is the quality of life, assessed by VAWS. In particular, we found that subjects with a WAI<37 had a lower score in the VAWS questionnaire and this score is associated with an increased risk of absenteeism. The correlations of the VAWS test with work disability have, to our knowledge, not yet been studied in literature. Therefore, because VAWS is a rapid test, less than a minute, our future proposal is to evaluate if it can be adopted in clinical practice to obtain more information on active work-related subjects with suspected OSA and, eventually, to evaluate post-treatment work improvements [25].

We must acknowledge certain limitations of our study. Firstly, our sample is based on referral to tertiary care centers and as such is not population based. Moreover, it included only patients of working age in employment for the previous 5 years who are, presumably, very sensitive to symptoms, like daytime sleepiness, which interfere with their activity. These recruitment setting and inclusion criteria may limit the external validity of our findings. Indeed, they might have contributed to a “selection” of a population where symptoms, particularly EDS, are very common, also in the no OSA group, possibly influencing the missing relevance of OSA in the multivariate analysis. Second, the etiology of EDS remains unclear in the no-OSA group. The sleepiness assessment used in this study was the subjective ESS questionnaire and no objective tests, as multiple sleep latency tests (MSLT), have been performed. The degree to which the ESS scores are correlated with the objective measurements of MSLT has generated much debate and conflicting results in the published literature, describing both no, weak, or positive associations [39,40]. Indeed, evidence of a clear relationship between ESS values and MSLT data has been reported in a cross-sectional study aimed to correlate subjective and objective sleepiness taking into account different confounding factors previously neglected, such as a limited sample size and the use of unsuitable tests for statistical analyses [40]. Although we acknowledge that MSLT could have characterized better daytime sleepiness in our study population, we are quite confident that ESS represents a reliable, easily administered, relatively simple and cost-effective method for evaluating this symptom. Finally the gender distribution of the present cohort was not balanced, women being under-represented. As different patterns of distribution of OSA symptoms have been reported in males and females, we cannot exclude that the under-representation of women might have influenced our results on work disability [41,42].

In conclusion, our results indicate that an older age, a decreased FEV_1_, an excessive daytime sleepiness and an impaired quality of life are independent risk factors of work disability in actively working subjects with suspected OSA. In the same subjects, a higher Charlson index and a lower quality of life increase the risk of sickness absences > 9 days.

Work disability and sickness absenteeism have implicit costs for workplaces related to reduced productivity and costs associated with compensating for an absent worker. Thus, to point out factors contributing to work disability and sickness absences in subjects with suspected OSA is warranted to adequately inform behavioral strategies in the individual and occupational levels.

## Supporting information

S1 File Study database used for statistical analysis. (XLS)
